# Effects of Sphingomonads on sugar beet growth and rhizosphere microbiota under continuous cropping

**DOI:** 10.3389/fmicb.2026.1793515

**Published:** 2026-03-05

**Authors:** Youkai Gao, Zenghao Wang, Jianan Cheng, Yihao Fu, Yuguang Wang, Yan Sun, Gui Geng, Yanchun Sun

**Affiliations:** College of Advanced Agriculture and Ecological Environment, Heilongjiang University, Harbin, China

**Keywords:** continuous cropping obstacles, microbial community structure, rhizosphere, Sphingomonads, sugar beet

## Abstract

**Introduction:**

Sugar beet is a crucial sugar crop, and its yield and quality are vulnerable to the adverse effects of continuous cropping. Plant growth-promoting rhizobacteria function as biological control agents and exhibit high potential for crop growth promotion.

**Methods:**

In this study, soil subjected to continuous sugar beet cropping was selected as the experimental substrate to evaluate the effects of *Sphingobium abikonense* strain W2, *Sphingomonas panni* strain W9, *Sphingomonas* sp. strain W13, and their mixed bacterial suspension on sugar beet seedling growth and soil properties using pot experiments. High-throughput sequencing was used to characterize changes in the rhizosphere soil microbial community structure.

**Results:**

The results indicated that Sphingomonads inoculation significantly improved the agronomic performance of sugar beet seedlings, as evidenced by increased plant height, stem diameter, aboveground and root fresh weight, and enhanced nitrogen and phosphorus uptake. In addition, inoculation increased soil pH, available potassium content, and sucrase activity. Microbial community analysis revealed that all inoculation treatments markedly altered the diversity and composition of the rhizosphere microbiome. Compared with the continuous cropping control, the inoculated soils exhibited a significantly higher abundance of Pseudomonadota, exceeding that observed under crop rotation. Moreover, beneficial genera (e.g., *Pseudomonas*, *Cupriavidus*, *Massilia*, and *Novosphingobium*) were enriched. Functional prediction demonstrated a significant enhancement of key metabolic processes, including ureolysis and xylanolysis.

**Conclusion:**

Overall, Sphingomonad inoculation effectively regulated the structure and function of the rhizosphere microbial community, improved soil enzyme activity and nutrient availability, and promoted sugar beet seedling growth. This study provides a theoretical foundation and potential biocontrol strategy for mitigating continuous cropping obstacles in sugar beet cultivation.

## 1 Introduction

Sugar beet (*Beta vulgaris* ssp. *vulgaris*) is an important temperate crop, with its roots contributing nearly 30% of global sugar production while also serving as a source of bioethanol and animal feed (Dohm et al., 2014; Li et al., 2022). Sugar beet exhibits broad climatic adaptability and is globally cultivated, with production systems predominantly concentrated in Europe, followed by Asia and North America (Bojović et al., 2022). Within Europe, sugar beet is the dominant raw material for commercial sugar production (Drennan, 1994). Owing to its considerable economic significance, sugar beet has been widely adopted and intensively cultivated. However, the long-term reliance on continuous cropping, defined as the repeated cultivation of the same crop or closely related species on identical land parcels, has been widely recognized as detrimental to soil quality (Ling et al., 2013). Accumulating evidence indicates that monoculture systems can drive substantial modifications in soil chemical properties, enzyme activity profiles, and the structure of bacterial communities (Liu et al., 2019; Chen et al., 2020). Concurrently, continuous cropping systems promote the accumulation of plant-derived autotoxins and the proliferation of harmful soil microorganisms, collectively increasing susceptibility to soil-borne diseases and constraining crop growth (Li et al., 2018). These interconnected soil degradation processes are collectively described as continuous cropping obstacles. Given its taproot architecture, deep rooting depth, and high nutrient demand, sugar beet is generally regarded as poorly suited for continuous cropping. As the duration of continuous cropping extends, agronomic performance progressively deteriorates, manifesting as reduced sugar accumulation, yield penalties, and an increased incidence of pests and diseases (Cui et al., 2022).

Various prevention and mitigation strategies have been developed to alleviate continuous cropping obstacles. Notably, crop rotation has been demonstrated to effectively suppress weeds and pests while enhancing soil nutrient use efficiency (Nevens and Reheul, 2001), thereby contributing to improvements in both crop yield and quality (Zhao et al., 2019). In addition, the application of organic fertilizers characterized by high levels of organic acids, bioactive peptides, and essential nutrients such as nitrogen, phosphorus, and potassium provides sustained support for crop growth by increasing soil organic matter, stimulating microbial activity, and improving soil physicochemical and biological properties (Wang T. et al., 2022; Liu et al., 2023). To counteract the accumulation of pathogenic microorganisms associated with long-term monoculture, soil sterilization approaches (e.g., high-temperature treatment and chemical fumigation) have been widely applied and shown to reduce pathogen loads effectively (Zhang et al., 2015). However, such practices may disrupt soil microecological balance and are increasingly viewed as incompatible with the principles of sustainable agriculture. Increasing studies have suggested that plants subjected to biotic stressors, such as pathogen infection, can actively modify the composition of root exudates to selectively recruit beneficial microorganisms with protective functions (Berendsen et al., 2018; Carrión et al., 2019; Liu et al., 2021). Accordingly, the isolation of functional microorganisms from natural environments and their development as antagonistic strains or plant growth-promoting rhizobacteria (PGPR) have emerged as effective biological strategies for disease suppression and crop growth enhancement (Morales-Cedeño et al., 2020). Sphingomonads are ubiquitous rhizosphere bacteria widely distributed in natural ecosystems and play pivotal roles in mutualistic plant–microbe interactions during plant growth (Sorouri et al., 2023). Taxonomically, they belong to the *α*-4 subclass of Proteobacteria and are characterized as aerobic gram-negative bacteria that typically form yellow colonies with smooth, glossy surfaces and well-defined margins. Morphologically, cells are predominantly short, rod-shaped, non-sporulating, and possess a single polar flagellum (White et al., 1996; Wang et al., 2019). Previous studies have demonstrated that numerous Sphingomonad strains exhibit dual functions related to plant growth promotion (PGP) and stress alleviation (Asaf et al., 2020). For instance, The *Sphingomonas* sp. strain CL01, isolated from the plant rhizosphere, significantly increased root dry weight, root length, and other growth indices of watermelon seedlings under continuous cropping conditions(Wang et al., 2015); Similarly, a strain isolated from Rosaceae plants was reported to secrete root-derived carbohydrates, thereby supplying additional nutrients and enhancing plant nutrient uptake efficiency (Takeuchi et al., 1995), while simultaneously exhibiting antagonistic activity against *Verticillium dahliae*, the causal agent of dahlia wilt (Gabriele and Ballin, 1994). Furthermore, Sphingomonad strains isolated from rice roots in California have been shown to inhibit major rice seedling pathogens and markedly promote seedling growth following inoculation (Adhikari et al., 2001). However, these studies are largely confined to crops such as rice and Rosaceae. The ecological functions and regulatory potential of Sphingomonads in the specific context of sugar beet continuous cropping remain unclear, representing a key knowledge gap this study aims to address.

Plant health is closely associated with the composition of rhizosphere microbial communities (Raaijmakers et al., 2008). In this context, inoculation with rhizobial agents derived from nitrogen-fixing bacteria has been shown to exert dual effects, simultaneously stimulating plant growth and reshaping the structure of core rhizosphere microbiota by strengthening microbial interactions (Zhong et al., 2019). However, the deliberate introduction of exogenous microorganisms can also generate unintended, non-target effects on indigenous microbial assemblages, with potential consequences for ecosystem biodiversity and functional stability (Mawarda et al., 2020). For example, the inoculation of *Sinorhizobium meliloti* L33 into alfalfa systems resulted in a marked reduction in the relative abundance of *Pseudomonas* in the rhizosphere, accompanied by a significant increase in rhizobial populations (Gui et al., 2023). Collectively, these observations underscore the significance of systematically evaluating the impact of microbial inoculants on rhizosphere community structure to achieve a comprehensive understanding of their non-target ecological effects.

Currently, 16S rRNA and ITS amplicon sequencing are widely applied as core techniques for profiling environmental microbial communities (Kui et al., 2021). To explore the potential of Sphingomonads in alleviating continuous cropping obstacles, this study combined pot experiments using three pre-screened strains with high-throughput amplicon sequencing. The objectives were to systematically assess the effects of inoculation on (1) the growth performance of sugar beet seedlings under continuous cropping conditions, (2) the physicochemical properties of rhizosphere soil, and (3) the composition and functional profile of the associated microbial community. Ultimately, this study aimed to elucidate the regulatory role of these bacterial treatments on key obstacle-associated microbial taxa and to predict functional shifts, thereby providing a theoretical basis for the development of targeted microecological management strategies for sugar beet cultivation.

## 2 Materials and methods

### 2.1 Materials and experimental design

The sugar beet cultivar ‘KWS1260’ (KWS Company, Einbeck, Germany) was used as the plant material. Three Sphingomonad strains (*Sphingobium abikonense* strain W2, *Sphingomonas panni* strain W9, and *Sphingomonas* sp. strain W13) and experimental soil were collected from the black soil region of Hulan District, Harbin City, Heilongjiang Province (46°00′14″E, 126°38′49″N). The soil had been subjected to two consecutive years of sugar beet cultivation and was used for pot experiments conducted in a growth chamber at the Agronomy Building of Heilongjiang University.

Li et al. (2024) demonstrated that under the cropping conditions in Hulan District, sugar beet plants exhibited significantly stunted growth and a higher incidence of disease in the second year of continuous cultivation. Therefore, soil exhibiting typical characteristics of continuous cropping obstacles was selected to investigate the potential of Sphingomonad strains to alleviate continuous-cropping stress in sugar beet. The three Sphingomonad strains used in this study were isolated in 2024 from soil samples collected from a sugar beet cultivation area at the experimental base in the Hulan District. Isolation was performed by screening on solid LB agar plates supplemented with streptomycin, followed by strain identification based on phenotypic traits, physiological and biochemical characteristics, and 16S rDNA gene sequence analysis (Weisburg et al., 1991). The corresponding 16S rDNA gene sequences were deposited in the NCBI GenBank database under the accession numbers PX849706, PX849724, and PX849743.

A pot experiment was conducted using six treatments. The control treatments included a positive control (NC), consisting of soil collected following the completion of a full three-year rotation cycle (sugar beet-corn-soybean); and a negative control (CK), consisting of soil from 2 years of continuous sugar beet cropping. In addition, four bacterial inoculation treatments were applied to the continuous cropping soil, including strain W2, strain W9, strain W13, and a mixture of the three strains [W(2 + 9 + 13)]. The bacterial suspension concentrations were 2.8 × 10^9^, 4.2 × 10^9^, and 1.0 × 10^8^ CFU/mL, and an equivalent mixed concentration, respectively. Each treatment was replicated eight times. The inoculation concentrations for each strain corresponded to their respective optimal levels for promoting plant growth, as determined in the preliminary experiments. In a preliminary experiment, the growth-promoting effects of Sphingomonad suspensions at different concentrations on sugar beet seedlings were evaluated by measuring key growth parameters. For each strain, the concentration that yielded the greatest promotion was selected for the subsequent experiments. Consequently, the inoculation concentrations differed among the three strains.

Each pot was filled with 900 g of uniformly prepared soil. After sowing with ‘KWS1260’ sugar beet seeds, five seedlings were retained per pot following emergence. During the growth period, plants were maintained under a light intensity of 700 μmol m^−2^ s^−1^ with a 14 h light/10 h dark photoperiod. Day and night temperatures were set at (28 ± 1)°C and (21 ± 1)°C, respectively, and the relative humidity was maintained at 40–50%. On days 7, 14, and 21 after sowing, 5 mL of the corresponding bacterial suspension was applied to each seedling by root irrigation into the rhizosphere (1–2 cm from the stem base) using a sterile syringe. For the mixed-bacterial treatment, the suspension consisted of equal volumes of the three selected strains, each applied at its predetermined optimal concentration.

### 2.2 Strain activation and preparation of bacterial suspension

Strains W2, W9, and W13, previously preserved at −80 °C, were removed from storage and thawed at room temperature. Under sterile conditions in a laminar flow hood, the strains were streaked onto LB solid medium using a sterilized inoculation loop, and the plates were incubated at 28 °C for recovery. Because the recovered strains were unstable at the initial stage, subculturing was performed prior to subsequent experiments.

LB plates exhibiting vigorous post-recovery growth were selected. Single colonies were aseptically picked and transferred onto fresh LB solid medium, followed by incubation at 28 °C for expanded cultivation.

Single colonies of strains W2, W9, and W13 were individually inoculated into LB liquid medium and cultured in a shaker at 28 °C and 180 rpm (Model ZWYR-2402, Shanghai Zhicheng Analysis Instrument Manufacturing Co., Ltd., Shanghai, China) for 24 h. The cultures were centrifuged at 5000 rpm(Model 5,418, Eppendorf AG, Hamburg, Germany), the supernatants were discarded, and the bacterial pellets were collected. The pellets were resuspended in an appropriate volume of sterile water to obtain bacterial suspensions, which were subsequently adjusted to the required concentration using a spectrophotometer.

### 2.3 Measurement of plant agronomic traits

Plants were harvested 28 days after sowing. Sugar beet plants with uniform growth were randomly sampled from each treatment, with eight biological replicates per treatment. Plant growth status was recorded. Plant height was measured using a measuring tape, and stem diameter was determined using a vernier caliper with a precision of 0.02 mm. The fresh weights of aboveground parts and roots were measured using an electronic analytical balance (Hangzhou Wante Weighing Apparatus Co., Ltd., model 5,001).

The total nitrogen content in the plant tissues was determined by distillation and titration of the digested samples using a Kjeldahl nitrogen analyzer. The total phosphorus content was measured using the molybdenum blue colorimetric method, and the total potassium content was determined using flame atomic absorption spectrometry.

### 2.4 Soil sample collection and processing

Soil sampling was performed on the 28th day after sowing. For non-rhizosphere soil, three sampling points were randomly selected within each treatment using a five-point sampling strategy, and the collected subsamples were combined to form a single biological replicate. The mixed soil samples were air-dried under natural conditions and subsequently sieved through a sterile 2 mm mesh prior to the analyses of soil physicochemical properties and enzyme activities.

For rhizosphere soil collection, three uniformly growing seedlings were randomly selected from each treatment. The roots were carefully excised using a sterile surgical blade and gently shaken to remove the loosely adhering bulk soil. The cleaned roots were transferred into sterile 50 mL centrifuge tubes containing 25 mL of sterile 10 mM PBS buffer (w/v) and agitated at 4 °C and 180 rpm(Model: ZWYR-2402, Shanghai Zhicheng Analysis Instrument Manufacturing Co., Ltd., Shanghai, China) for 5 h to detach the rhizosphere soil. Following root removal, the resulting suspension was centrifuged at 3200 × g for 15 min at 4 °C using a benchtop low-speed centrifuge (model TDZ5-WS), and the pellet representing rhizosphere soil was collected. Each treatment had five biological replicates. All soil samples were stored at −80 °C until subsequent high-throughput sequencing analysis.

### 2.5 Soil physicochemical properties and enzyme activity analysis

The soil ammonium nitrogen (NH_4_^+^-N) and nitrate nitrogen (NO_3_^−^-N) contents were determined using the magnesium oxide–Devarda alloy distillation method, involving the reduction of nitrate to ammonia via alkaline distillation in a Kjeldahl apparatus, followed by absorption in boric acid and quantification by automatic titration. Available phosphorus (AP) was extracted with 0.5 mol·L^−1^ NaHCO₃ (pH 8.5) and quantified by the molybdenum-antimony anti-colorimetric method, which is based on the formation of a phosphomolybdenum blue complex. Available potassium (AK) and micronutrients were extracted with 1.0 mol·L^−1^ ammonium acetate, with AK determined by flame photometry and micronutrients (e.g., Fe, Mn, Cu, Zn) by atomic absorption spectrometry. Soil pH was measured using a potentiometer (Shanghai Jingke Instrument Co., Ltd.) after extraction at a water-to-soil ratio of 5:1 (Lu, 2005).

Soil enzyme activities were assessed as follows. Urease activity was determined colorimetrically by measuring ammonia released from urea hydrolysis (indophenol blue method). Phosphatase activity was assayed based on phenol release from disodium phenyl phosphate, quantified colorimetrically. Sucrase activity was measured by detecting reducing sugars produced from sucrose hydrolysis using the 3,5-dinitrosalicylic acid (DNS) method. Catalase activity was determined by titrating residual H₂O₂ with KMnO₄ after enzymatic decomposition (Lu, 2005). All soil samples were analyzed in triplicate, and corresponding inorganic controls were included during enzyme activity assays.

### 2.6 High-throughput sequencing and bioinformatics analysis

Total microbial DNA was extracted from the soil samples using the E.Z.N.A.^®^ Soil DNA Kit (Omega Bio-tek, Norcross, GA, USA). DNA quality was evaluated by 1% agarose gel electrophoresis, and DNA concentration and purity were determined using a NanoDrop 2000 spectrophotometer (Thermo Fisher Scientific, USA). The V3–V4 region of the bacterial 16S rRNA gene was amplified using primers 338F and 806R, and the fungal ITS1 region was amplified using primers ITS1F and ITS2R. The PCR products were excised from 2% agarose gels and purified using a DNA gel recovery and purification kit (PCR Clean-Up Kit, China Yuhua). Amplicon concentrations were quantified using a Qubit 4.0 fluorometer (Thermo Fisher Scientific, USA). The purified amplicons were subjected to paired-end sequencing on an Illumina NovaSeq 2000 platform by Shanghai Majorbio Bio-pharm Technology Co., Ltd.

The raw sequencing reads were quality-filtered using fastp (Chen et al., 2018) (https://github.com/OpenGene/fastp, version 0.19.6). The paired-end reads were merged and low-quality bases were trimmed using FLASH (Magoč and Salzberg, 2011) (http://www.cbcb.umd.edu/software/flash, version 1.2.11). The DADA2 plugin (Edgar, 2013) within the QIIME 2 pipeline (Barberán et al., 2012) was subsequently applied to denoise the filtered sequences and generate high-quality amplicon sequence variants (ASVs). The taxonomic assignment of ASVs was conducted using the Vsearch classifier in QIIME 2, based on the Silva database (v138) for bacteria and the UNITE database (v8.0) for fungi. To reduce the influence of uneven sequencing depth among samples, sequences assigned to chloroplasts and mitochondria were eliminated, followed by rarefaction to normalize the dataset (Li et al., 2020).

### 2.7 Statistical analysis

Plant and soil parameters were analyzed using one-way ANOVA with IBM SPSS 26 (SPSS Inc., Somers, USA). Multiple comparisons were performed using Tukey’s test at a significance level of *p* < 0.05. Figures were generated using GraphPad Prism software.

The microbial alpha diversity indices were calculated using Mothur^1^ (Schloss et al., 2009). Differences among groups were evaluated using the Wilcoxon rank-sum test. Principal coordinate analysis (PCoA) based on Bray–Curtis distances (Xiong et al., 2020) was employed to visualize the similarities in microbial community structure, and PERMANOVA was adopted to test the significance of intergroup variation. The LEfSe analysis^2^ (LDA > 3, *p* < 0.05) (Segata et al., 2011) was applied to identify the bacterial taxa from phylum to genus levels presenting significant differences in relative abundance among treatments. Distance-based redundancy analysis (db-RDA) was performed to examine the effects of soil physicochemical factors on microbial community structure. Spearman correlation analysis (|r| > 0.6, *p* < 0.05) was used to identify key taxa and construct correlation networks. The functional profiles of the bacterial and fungal communities were predicted using the FAPROTAX and FUNGuild databases, respectively. All microbial sequencing data processing and analyses were conducted using the Majorbio Cloud Platform.^3^

## 3 Results

### 3.1 Soil properties and agronomic performance

The effects of different Sphingomonad suspension treatments on the growth parameters and nutrient contents of sugar beet seedlings are summarized in Table 1. With respect to growth performance, the CK group (continuous cropping soil) showed significant reductions in plant height (Height), stem diameter (SD), aboveground fresh weight (FW), and root fresh weight (RFW) compared with the NC group (crop rotation soil). The application of bacterial suspensions to continuous cropping soil markedly alleviated continuous cropping constraints. All inoculation treatments presented significantly higher growth indices than those observed in the CK group and, in some cases, exceeded those of the NC group. Specifically, plant height in the W2, W9, W13, and mixed [W(2 + 9 + 13)] treatments increased by 45.45, 44.73, 41.56, and 38.24%, respectively, compared with CK. Similarly, RFW increased by 137.93, 148.27, 127.59, and 148.27%, respectively, with no significant differences detected among the bacterial suspension treatments.

**Table 1:** Plant growth indicators and nutrient content measurement results.

Grope	NC	CK	W2	W9	W13	W(2 + 9 + 13)
Height (cm)	8.30 ± 0.72b	6.93 ± 1.20c	10.08 ± 1.41a	10.03 ± 1.29a	9.81 ± 1.24a	9.58 ± 0.96a
SD (cm)	2.59 ± 0.35b	1.86 ± 0.34c	3.25 ± 0.56a	3.32 ± 0.57a	3.35 ± 0.48a	3.30 ± 0.48a
FW (g)	0.94 ± 0.17b	0.61 ± 0.19c	1.14 ± 0.32ab	1.24 ± 0.36ab	1.43 ± 0.40a	1.31 ± 0.34a
RFW (g)	0.59 ± 0.17a	0.29 ± 0.07b	0.69 ± 0.23a	0.72 ± 0.30a	0.66 ± 0.29a	0.72 ± 0.29a
DN (mg/g)	88.07 ± 0.51 cd	84.00 ± 1.87d	88.58 ± 2.04 cd	91.93 ± 1.40c	139.49 ± 3.05a	121.67 ± 8.65b
RDN (mg/g)	174.30 ± 7.70b	101.42 ± 2.90f	120.82 ± 0.59e	149.89 ± 0.85c	186.74 ± 1.78a	133.38 ± 4.66d
DP (mg/g)	1.43 ± 0.24a	0.95 ± 0.23b	0.75 ± 0.28bc	1.39 ± 0.01a	0.40 ± 0.09c	0.49 ± 0.17c
RDP (mg/g)	2.33 ± 0.36a	1.35 ± 0.22c	1.59 ± 0.12bc	1.83 ± 0.09b	1.71 ± 0.17bc	1.43 ± 0.06c
DK (mg/g)	10.30 ± 0.53a	3.35 ± 0.16d	2.96 ± 0.25d	3.24 ± 0.67d	7.95 ± 1.30b	5.40 ± 1.23c
RDK (mg/g)	9.11 ± 0.38a	7.13 ± 0.12b	6.33 ± 0.29c	6.64 ± 0.36c	9.22 ± 0.16a	5.84 ± 0.20d

Height, plant height; SD, stem diameter; FW, aboveground fresh weight; RFW, root fresh weight; DN, aboveground total nitrogen; RDN, root total nitrogen; DP, aboveground total phosphorus; RDP, root total phosphorus; DK, aboveground total potassium; RDK, root total potassium. Values are presented as mean ± standard deviation (*n* = 5). Different lowercase letters within the same row indicate statistically significant differences at *p* < 0.05, according to Duncan’s multiple range test. NC, soybean rotation soil; CK, soil from 2 years of continuous sugar beet cropping; W2, two-year continuous cropping soil inoculated with strain W2; W9, two-year continuous cropping soil inoculated with strain W9; W13, two-year continuous cropping soil inoculated with strain W13; W(2 + 9 + 13), two-year continuous cropping soil treated with a mixture of the three strains. The same abbreviations are used in the subsequent sections.

In terms of plant nutrient status, CK exhibited significant decreases in root total nitrogen (RDN), aboveground total phosphorus (DP), root total phosphorus (RDP), aboveground total potassium (DK), and root total potassium (RDK) compared with NC. Distinct bacterial suspensions demonstrated functional differentiation in mitigating continuous cropping effects. The total nitrogen content, including both aboveground and root nitrogen, was significantly higher in plants treated with strains W2, W9, and W13 and the mixed suspension [W(2 + 9 + 13)] than in the CK group, indicating enhanced nitrogen uptake under continuous cropping conditions. In particular, strain W13 produced the strongest promotive effect, with both aboveground and root nitrogen contents significantly exceeding those of the other treatments, and it also contributed to improved potassium uptake. Moreover, W9 treatment enhanced phosphorus absorption in continuously cropped sugar beet. Overall, Sphingomonad suspension treatments significantly promoted seedling growth and nutrient acquisition in continuously cropped sugar beet, thereby effectively alleviating continuous cropping obstacles.

The application of the three Sphingomonad suspensions, either individually or in combination, induced pronounced changes in the physicochemical properties and enzyme activities of soils subjected to continuous cropping (Table 2). Compared with the NC, the CK group exhibited a significant decline in soil pH, indicating clear soil degradation. In contrast, inoculation with strains W2, W9, and W13, as well as their mixed treatment [W(2 + 9 + 13)], resulted in a significant elevation of soil pH compared with CK, thereby partially mitigating the continuous cropping-induced acidification, while the pH levels remained lower than those observed in the NC group.

**Table 2:** Soil physicochemical properties and enzyme activities under different treatments.

Grope	NC	CK	W2	W9	W13	W(2 + 9 + 13)
pH	6.64 ± 0.05a	6.04 ± 0.06e	6.52 ± 0.04b	6.43 ± 0.00c	6.18 ± 0.06d	6.47 ± 0.03bc
AN (NH_4_) (mg/kg)	19.60 ± 1.85a	16.10 ± 1.85ab	12.36 ± 2.14b	12.25 ± 2.45b	15.40 ± 2.52b	11.20 ± 1.40c
AP (mg/kg)	75.88 ± 8.75b	99.64 ± 9.02a	101.02 ± 5.32a	104.43 ± 14.46a	93.25 ± 4.05a	95.27 ± 4.64a
AK (mg/kg)	171.80 ± 1.84c	202.44 ± 1.61b	201.52 ± 3.46b	216.73 ± 3.92a	215.35 ± 11.75a	202.90 ± 1.61b
Catalase (μmol H_2_O_2_·d^-1^·g^-1^)	102.35 ± 2.85a	100.43 ± 2.61a	104.75 ± 2.55a	104.15 ± 3.43a	103.95 ± 5.84a	105.83 ± 0.94a
Phosphatase (μmol phenol· d^-1^·g^-1^)	73.93 ± 1.65b	63.20 ± 2.11c	60.93 ± 2.42c	75.80 ± 3.73b	84.91 ± 2.09a	74.92 ± 3.05b
Urease (μg NH_3_-N·d^-1^·g^-1^)	1.57 ± 0.10d	1.66 ± 0.07c	1.82 ± 0.03b	1.90 ± 0.05a	1.62 ± 0.06 cd	1.57 ± 0.02d
Sucrase (mg glucose·d^-1^·g^-1^)	26.06 ± 0.22c	23.87 ± 0.56c	30.72 ± 1.03b	33.87 ± 2.44a	35.51 ± 0.89a	33.48 ± 1.37a

pH, acidity–alkalinity; AN, ammonium nitrogen; AP, available phosphorus; AK, available potassium. Values are presented as mean ± standard deviation (*n* = 5). Different lowercase letters within the same row indicate statistically significant differences at *p* < 0.05, according to Duncan’s multiple range test.

In terms of soil nutrient status, CK demonstrated a non-significant decrease in AN content compared with NC, whereas AP and AK contents were significantly higher. Following the bacterial suspension application, the AN content generally declined, with the mixed treatment exhibiting a significant reduction of 30.43% compared with CK. No significant differences in AP content were detected between the bacterial suspension treatments and CK, whereas all values were significantly higher than those observed in NC. The W9 and W13 treatments significantly increased soil AK content. Although the mixed treatment presented higher AK levels than CK, the difference was not statistically significant. Overall, the trend in AK content across treatments followed the order: W9 > W13 > W(2 + 9 + 13) > CK > W2 > NC.

Soil enzyme activities responded to Sphingomonad inoculation in an enzyme-specific manner. Catalase activity did not differ significantly among the six treatments. In contrast, phosphatase activity was significantly enhanced by all treatments except W2, with W13 increasing phosphatase activity by 34.36%, exceeding the level observed in the NC group. Urease activity exhibited evident strain-dependent responses. It was significantly increased by W9 and W2 but reduced by the mixed treatment, with the activity ranking as follows: W9 > W2 > CK > W13 > W(2 + 9 + 13) = NC. No significant difference in sucrase activity was detected between CK and NC, whereas all bacterial suspension treatments significantly enhanced sucrase activity compared with CK, with enhancement rates ranging from 28.70 to 48.76%.

### 3.2 Alpha diversity of microbial communities

Following quality control of the high-throughput sequencing data, a total of 1,800,425 high-quality reads were obtained for the bacterial 16S rRNA gene (V3-V4 region), averaging 50,012 ± 4,545 reads per sample. For the fungal ITS1 region, 1,687,328 high-quality reads were obtained, with a mean of 46,870 ± 4,173 reads per sample. Specifically, the sequencing depth ranged from 43,444 to 60,440 reads per sample for bacteria and from 48,789 to 65,527 reads per sample for fungi, demonstrating uniform and sufficient coverage across all samples. This depth provided a robust foundation for the subsequent microbial community analyses.

**Figure 1:**
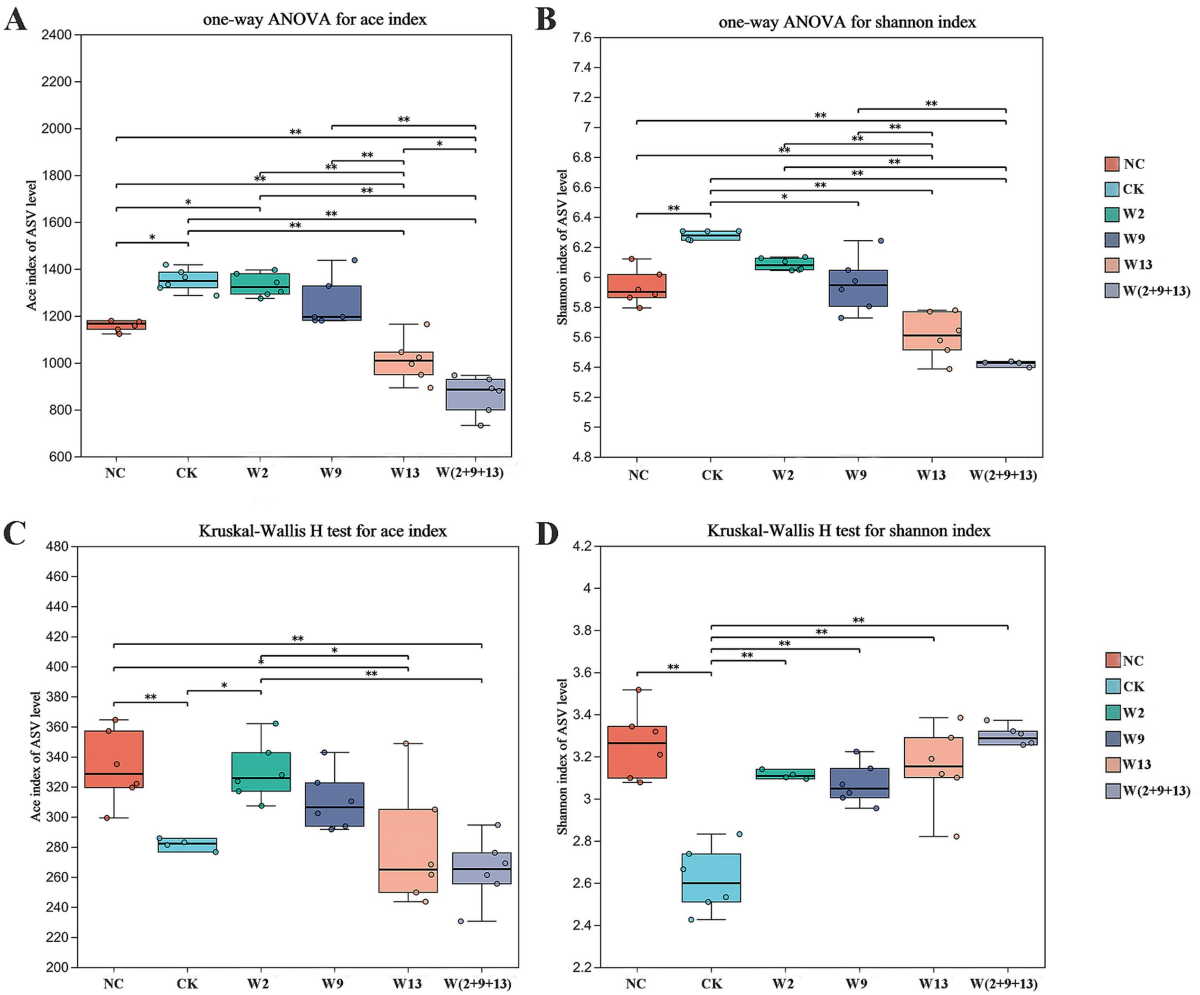
Alpha diversity of bacterial and fungal communities. Panels **(A,B)** show the alpha diversity of bacterial communities, and panels **(C,D)** show the alpha diversity of fungal communities. Panels **(A,C)** present the Ace index, and panels **(B,D)** present the Shannon index. Asterisks (*) indicate statistically significant differences at *p* < 0.05.

Alpha diversity integrates both species richness and evenness within microbial communities. In this study, the Ace index was adopted to characterize species richness, while the Shannon index was employed to evaluate the overall community diversity. Alpha diversity analyses were performed on the rhizosphere soil microbial communities of sugar beet under different treatments (Figure 1).

Compared with the CK group, both NC and Sphingomonad inoculation treatments exhibited reduced bacterial community richness (Ace index) and diversity (Shannon index) in the rhizosphere soil, with most differences being statistically significant (Figures 1A,B). These results indicate that Sphingomonad application decreased bacterial alpha diversity in the rhizosphere. The magnitude of this reduction followed the order W(2 + 9 + 13) > W13 > W9 > W2. Additionally, bacterial richness and diversity in the W13 and W(2 + 9 + 13) treatments were lower than those observed in the NC group.

For rhizosphere fungal communities (Figures 1C,D), CK demonstrated significantly lower richness and diversity than NC. All bacterial suspension treatments significantly increased fungal community diversity compared with CK, approaching the levels observed in NC. For richness, only the W2 treatment resulted in a significant increase compared with CK and reached a level similar to that of NC, whereas the other three inoculation treatments presented a slightly higher richness than CK without statistical significance. Overall, Sphingomonad application markedly enhanced rhizosphere fungal diversity and increased fungal richness.

In summary, Sphingomonad inoculation reshaped the rhizosphere microbial community structure in continuously cropped sugar beet soil by reducing bacterial alpha diversity while promoting fungal diversity.

### 3.3 Beta diversity of microbial communities

**Figure 2:**
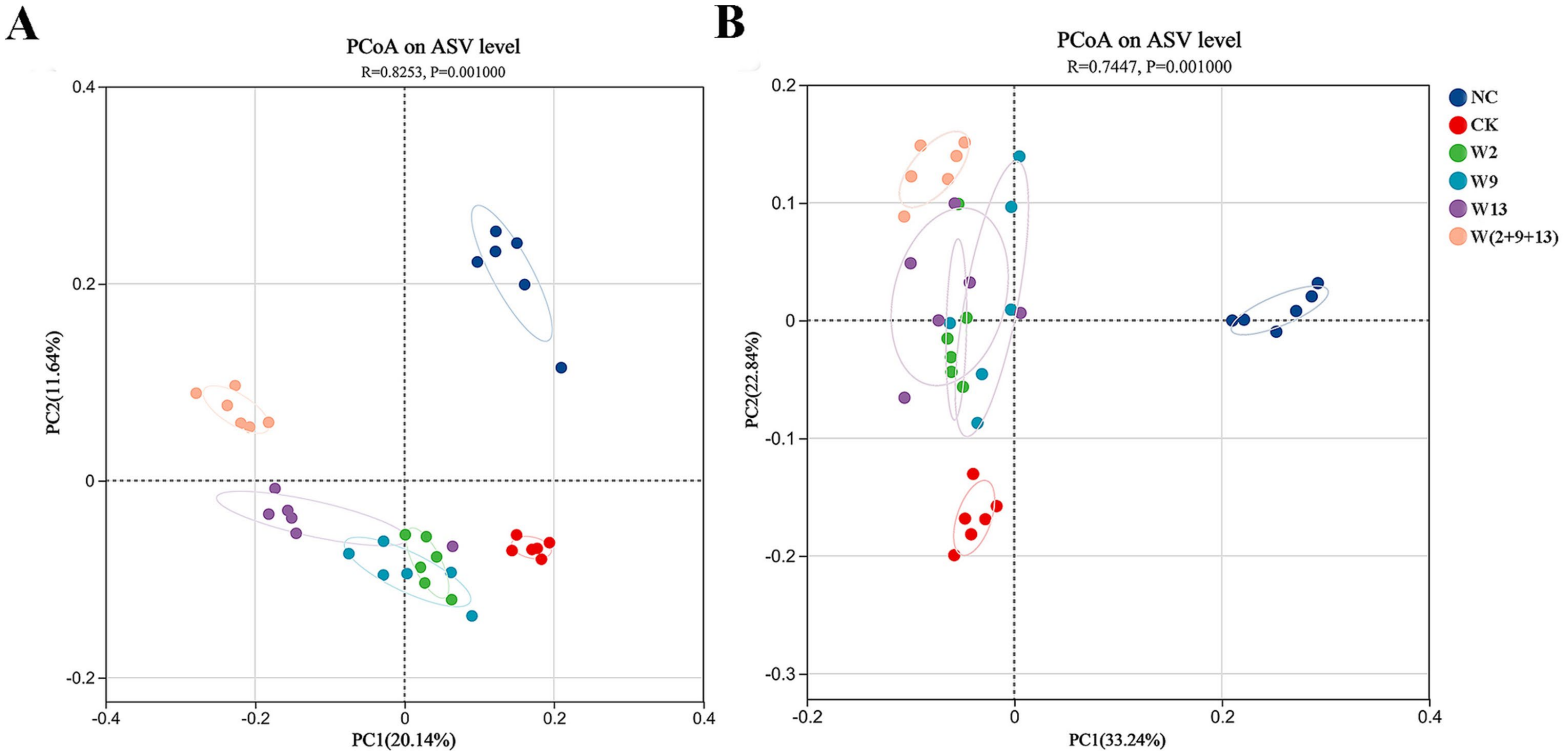
Principal coordinate analysis (PCoA) of bacterial and fungal communities based on Bray–Curtis distance **(A)** Bacterial community and **(B)** fungal community.

Principal coordinate analysis (PCoA) based on Bray–Curtis distances was conducted to evaluate the effects of different treatments on the microbial community structure. The results suggested that all treatments significantly altered both bacterial and fungal community structures, although distinct response patterns were observed (Figure 2).

For bacterial communities (Figure 2A), sample variation was primarily distributed along the PC2 axis. The samples from NC and W(2 + 9 + 13) treatment were located on the positive half-axis and formed distinct clusters. In contrast, the samples from the CK and the three single-strain treatments (W2, W9, and W13) clustered on the negative half-axis. Notably, samples from the three single-strain treatments were highly aggregated and clearly separated from the CK group. These results indicate that the application of Sphingomonads substantially reshaped the bacterial community structure, with the mixed suspension treatment exerting the strongest effect and producing community compositions that differed significantly from both the crop rotation control and the single-strain treatments.

For fungal communities (Figure 2B), compositional variation was mainly explained by the PC1 axis. The NC group was distinctly separated on the right side, whereas the CK and all bacterial suspension treatments clustered on the left. Further analysis revealed that the W(2 + 9 + 13) treatment formed an independent cluster within this region and was clearly separated from CK. The three single-strain treatments also clustered closely together and were distinct from CK. These results demonstrated that Sphingomonad application induced pronounced shifts in fungal community structure, with the mixed treatment showing a unique and more differentiated effect.

### 3.4 Shifts in microbial community structure

**Figure 3:**
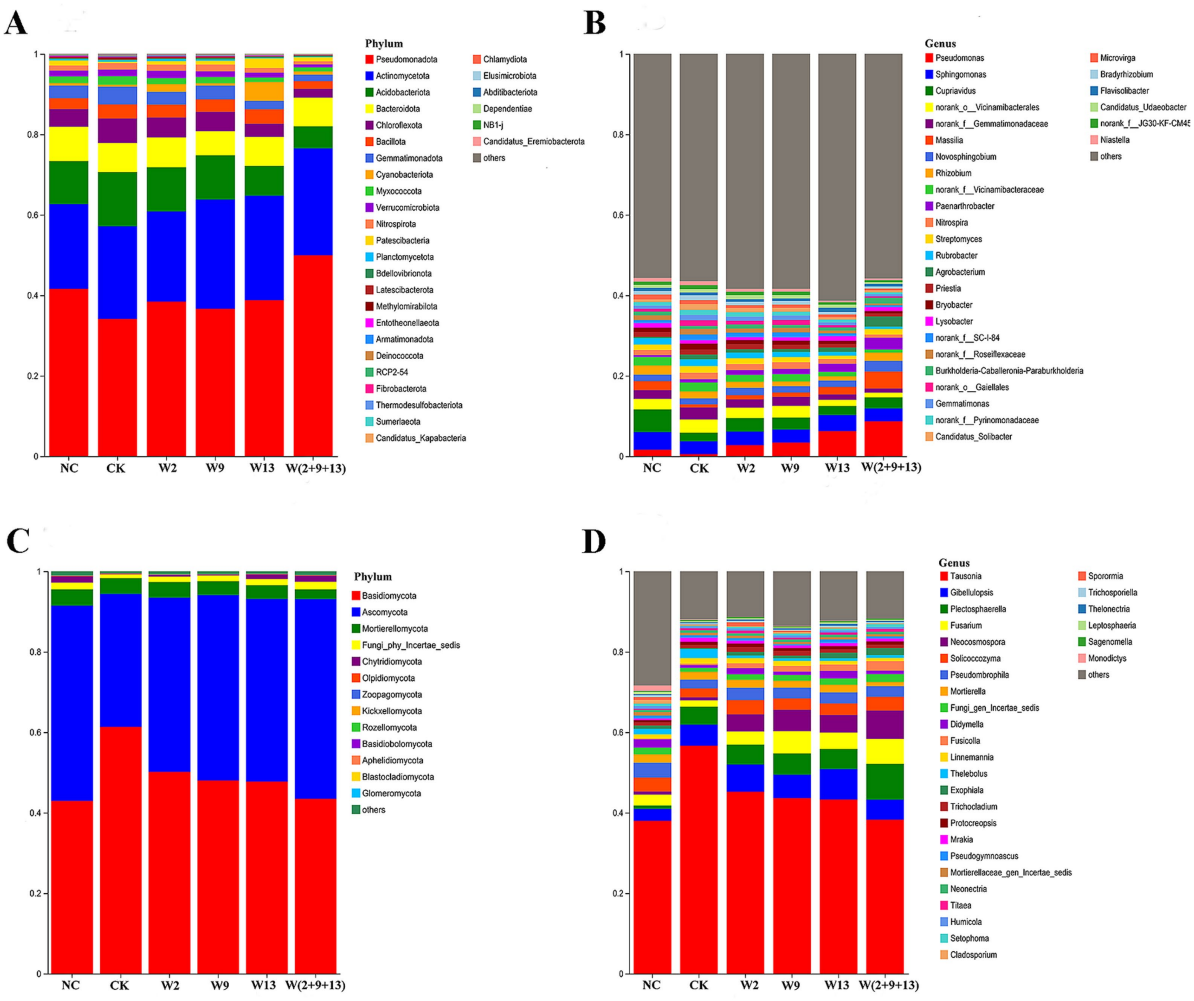
Relative abundance of microbial communities in sugar beet rhizosphere soil at the phylum and genus levels under different treatments. **(A)** Bacterial phyla, **(B)** bacterial genera, **(C)** fungal phyla, and **(D)** fungal genera. Different colors indicate distinct bacterial or fungal taxa at corresponding taxonomic levels.

To further elucidate the influence of Sphingomonad application on the rhizosphere microbiome of sugar beet, the changes in bacterial and fungal community composition were examined at the phylum and genus levels across treatments (Figure 3).

At the bacterial phylum level (Figure 3A), Pseudomonadota (formerly Proteobacteria), Actinomycetota, Acidobacteriota, Bacteroidota, and Chloroflexota dominated the communities, accounting for more than 85% of the total relative abundance. Notably, Pseudomonadota was the most abundant phylum, with relative abundance ranging from 34.11 to 49.93%. Compared with CK, both the single-strain and mixed bacterial suspension treatments increased the abundance of Pseudomonadota, with the largest increase observed in the W(2 + 9 + 13) treatment, where the abundance exceeded that of NC. Moreover, the W13 and mixed treatments reduced the relative abundances of Acidobacteriota and Gemmatimonadota, whereas Cyanobacteriota and Patescibacteria reached the highest abundances in the W13 treatment, at 4.7 and 2.37%, respectively. No significant differences were detected among treatments for the remaining phyla.

At the bacterial genus level (Figure 3B), the dominant genera included *Pseudomonas*, *Sphingomonas*, *Cupriavidus*, *Massilia*, *Novosphingobium*, and *Rhizobium*. The CK group exhibited the lowest relative abundance of *Pseudomonas*. In contrast, all Sphingomonad suspension treatments significantly increased the *Pseudomonas* abundance compared with CK, with the mixed treatment presenting the greatest increase, reaching 8.69%. In the NC, *Cupriavidus* and *Sphingomonas* had the highest abundance levels, and all bacterial suspension treatments further enhanced the relative abundances of these genera compared to CK. Additionally, the mixed treatment significantly increased the relative abundances of *Massilia*, *Novosphingobium*, and *Paenarthrobacter* to 4.25, 2.65, and 2.94%, respectively.

At the fungal phylum level (Figure 3C), Basidiomycota and Ascomycota dominated the community, accounting for nearly 90% of the total relative abundance. Basidiomycota exhibited the highest relative abundance in the CK (61.36%) and the lowest in the NC (42.92%), whereas Ascomycota displayed the opposite pattern, with the lowest abundance in CK (33.02%) and the highest in the mixed bacterial suspension treatment (49.67%). Compared with CK, the Sphingomonad suspension treatments consistently decreased the relative abundance of Basidiomycota while increasing that of Ascomycota and promoting Chytridiomycota to varying extents. Notably, W13 and W(2 + 9 + 13) treatments produced community patterns comparable to those observed in NC.

At the fungal genus level (Figure 3D), dominant genera included *Tausonia*, *Gibellulopsis*, *Plectosphaerella*, *Fusarium*, and *Neocosmospora*. *Tausonia* had the highest relative abundance in CK, and its proportion was significantly reduced following bacterial suspension application. In contrast, Sphingomonads treatments increased the relative abundances of *Fusarium* and *Neocosmospora* to varying degrees, with the magnitude of enhancement following the order W(2 + 9 + 13) > W9 > W13 > W2, with the mixed treatment exhibiting the strongest effect. In addition, all bacterial suspension treatments significantly suppressed the relative abundance of *Thelebolus*.

#### 3.4.1 Analysis of biomarker differences between treatments

**Figure 4:**
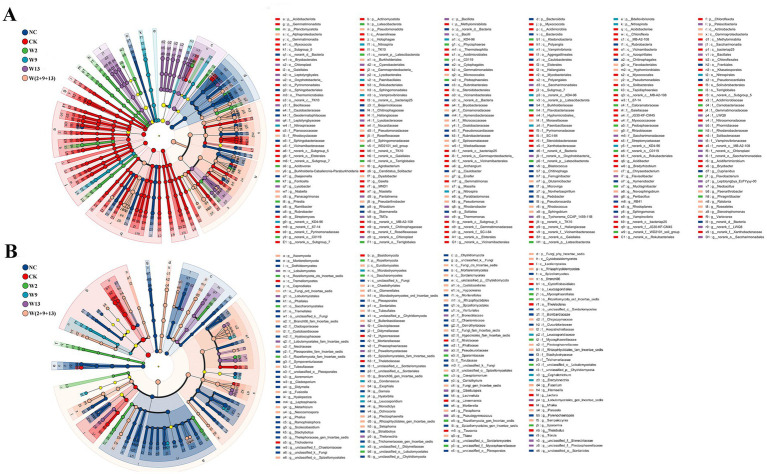
Cladograms generated by LEfSe analysis illustrating differences in the relative abundance of bacteria **(A)** and fungi **(B)** across treatment groups. Taxa are displayed from the phylum to the genus level. The concentric rings, from inside to outside, represent successive taxonomic ranks (phylum, class, order, family, and genus). Colored nodes indicate microbial taxa that serve as significant biomarkers for the communities represented by corresponding colors. The legend on the right lists the taxa corresponding to the letters shown in the figure.

The LEfSe approach (LDA > 3) was applied to identify the differences in rhizosphere microbial communities among soil treatments (Figure 4). Cladogram analysis indicated that the number of significantly differentiated biomarker taxa was higher for bacteria than for fungi. Within the bacterial community, the CK group covered the largest area and contained the highest number of nodes, demonstrating that a broader range of bacterial taxa participated in key biological processes under this condition. In contrast, within the fungal community, the NC group exhibited the largest area and greatest number of nodes, followed by the W(2 + 9 + 13) treatment, whereas the CK and W9 treatments displayed the fewest nodes. These patterns suggest that the NC and W(2 + 9 + 13) treatments harbored a greater number of fungal taxa associated with key biological functions, and that the mixed bacterial suspension treatment increased the abundance of fungal biomarker taxa in continuously cropped soil.

Further LEfSe analysis of the rhizosphere bacterial community (Figure 4A) revealed pronounced differences in the dominant taxa among treatments. At the phylum level, Acidobacteriota, Actinomycetota, Chloroflexota, Pseudomonadota, Bacteroidota, Bacillota, and Myxococcota were relatively abundant and exhibited distinct distribution patterns across the six treatments. In the.

NC group, Bacteroidota played a key functional role at the phylum level, and NC showed significant enrichment of *Rubrobacter* (class to genus), *Caulobacter* (order to genus), *Pseudonocardia* (order to genus), *Ramlibacter* (genus), *Rhizobium* (genus), and *Skermanella* (genus), all of which are associated with important biological functions. In the CK group, the predominant phyla included Acidobacteriota, Gemmatimonadota, Actinomycetota, Chloroflexota, and Myxococcota. At the genus level, *Streptomyces*, *Bryobacter*, and *Archangium* were significantly enriched. Notably, the application of Sphingomonads to continuously cropped soil substantially reduced the abundance of the dominant bacterial taxa. Distinct biomarker assemblages were identified for each bacterial suspension treatment. In the W2 treatment, rhizosphere biomarkers mainly comprised Planctomycetota and Latescibacterota (phylum), C0119 (class to genus), *Acidovorax* (genus), *Mucilaginibacter* (genus), *Fonticella* (family to genus), *Flavobacterium* (family to genus), and *Priestia* (genus). In the W9 treatment, biomarkers were dominated by *Nitrospira* (phylum to genus), Bdellovibrionota, KD4-96 (class to genus), Vampirovibrio (class to order), and Paenibacillaceae (order to family). In the W13 treatment, significant enrichment was observed for Bacillota, Lysobacterales (order), *Pantalinema* (order to genus), *Leptolyngbya* (order to genus), and *Flavisolibacter* (genus). The W(2 + 9 + 13) treatment exhibited the greatest diversity in biomarker taxa. At the phylum level, dominant biomarkers included Pseudomonadota, Gammaproteobacteria, and Actinobacteria. At the genus level, key enriched taxa were *Sphingomonas* (order to genus), *Dyadobacter* (family to genus), *Massilia* (genus), and *Rhodococcus* (genus).

In the rhizosphere fungal community (Figure 4B), Ascomycota and Basidiomycota were the two dominant phyla, and several taxa within these groups differed significantly among the six treatments. In the NC group, the key fungal taxa included Chytridiomycota, Dothideomycetes (class), *Sordariomycetes* (order to genus), *Pleosporales* (order to genus), *Mortierella* (phylum to genus), *Cladosporium* (order to genus), and *Gamsia* (genus). In the CK group, fungi such as *Trichoderma* (family and genus), *Tausonia* (genus), *Linnemannia* (genus), *Mrakia* (genus), and *Thelebolus* (genus) were identified as the major taxa associated with key biological functions. Fungal biomarker assemblages varied significantly among the bacterial suspension treatments. In the W2 treatment, significant enrichment was observed for taxa including *Mycosphaerellaceae* (order to genus), *Sporormia* (family and genus), and *Lobulomycetales* (family and genus). The W9 treatment was characterized by enrichment of *Condenascus* (genus), *Hyalorbilia* (genus), *Leucosporidium* (genus), and *Cephalotrichum* (genus). In the W13 treatment, the key biomarker taxa comprised Bionectriaceae (family), *Thelonectria* (genus), *Pseudogymnoascus* (genus), Powellomycetaceae (genus), and *Gibellulopsis* (genus). In the W(2 + 9 + 13) treatment, the enriched taxa included *Neocosmospora* (class to genus), *Fusicolla* (class to genus), *Plectosphaerella* (class to genus), *Titaea* (order to genus), *Sampaiozyma* (order to genus), *Hannaella* (family and genus), *Parasola* (genus), and *Metarhizium* (genus), all of which were associated with important biological functions. Overall, the introduction of Sphingomonads altered the composition of fungal biomarker taxa in the sugar beet rhizosphere to varying degrees.

### 3.5 Environmental drivers of microbial community composition

**Figure 5:**
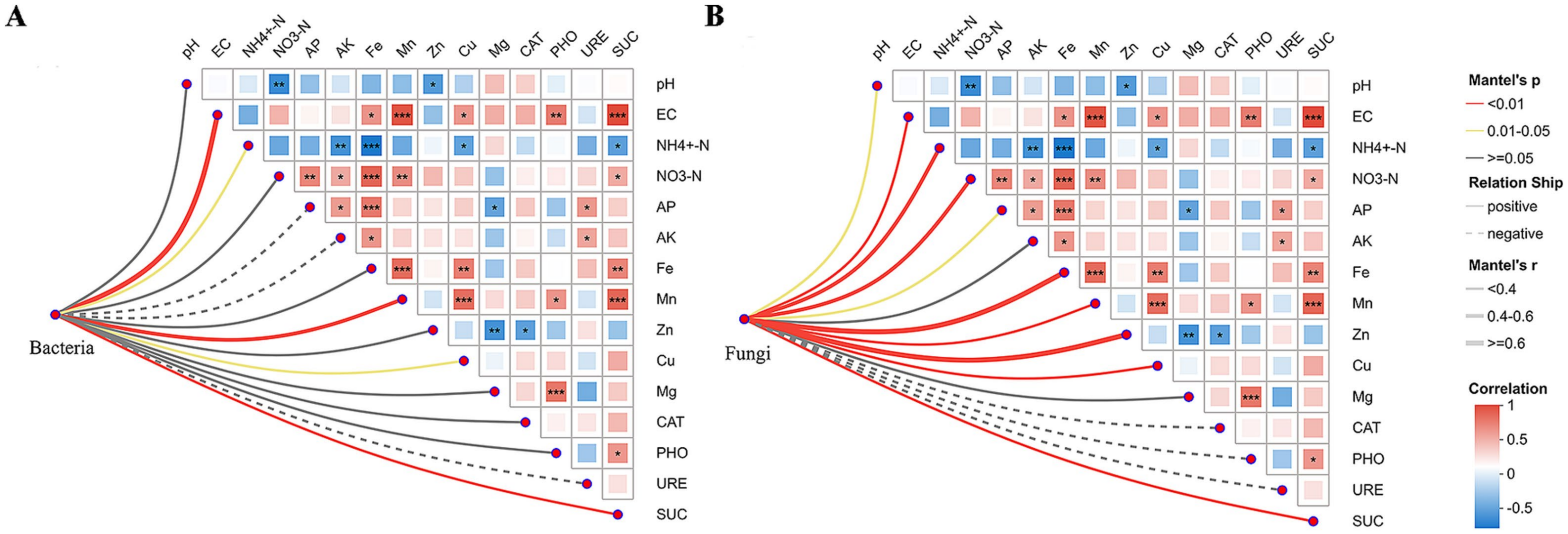
Mantel test analysis identifying key environmental factors associated with bacterial and fungal community structures. Panels **(A,B)** show Pearson correlations between environmental factors and bacterial **(A)** and fungal **(B)** community structures, respectively, based on Mantel tests. Solid lines indicate positive correlations, and dashed lines indicate negative correlations. The color intensity of the squares reflects the magnitude *of the P*earson correlation coefficient. Significance levels are denoted by asterisks: *0.01 < *p* ≤ 0.05, **0.001 < *p* ≤ 0.01, and ***p ≤ 0.001.

Mantel tests were used to quantify the impact of environmental factors on soil microbial community structure. Overall, fungal community assembly was associated with a more complex environmental driver network than bacterial communities (Figure 5). Among the soil physicochemical properties, EC, ammonium nitrogen (NH_4_^+^-N), copper (Cu), and manganese (Mn) were significantly correlated with the bacterial community structure (*p* < 0.05), with EC and Mn exhibiting the strongest associations (*p* < 0.01). In contrast, the fungal community composition was significantly influenced by a broader suite of variables, including pH, EC, NH_4_^+^-N, NO_3_^−^-N, AP, AK, Mn, zinc (Zn), and Cu, among which Zn and Fe exhibited the strongest correlations. Regarding soil extracellular enzyme activities, both bacterial and fungal community structures were strongly and positively correlated with sucrase (SUC) activity (*p* < 0.01), indicating a close link between microbial community composition and SUC dynamics. Collectively, these results demonstrated that the diversity and composition of rhizosphere fungal communities were more tightly coupled to environmental factors than those of bacterial communities.

**Figure 6:**
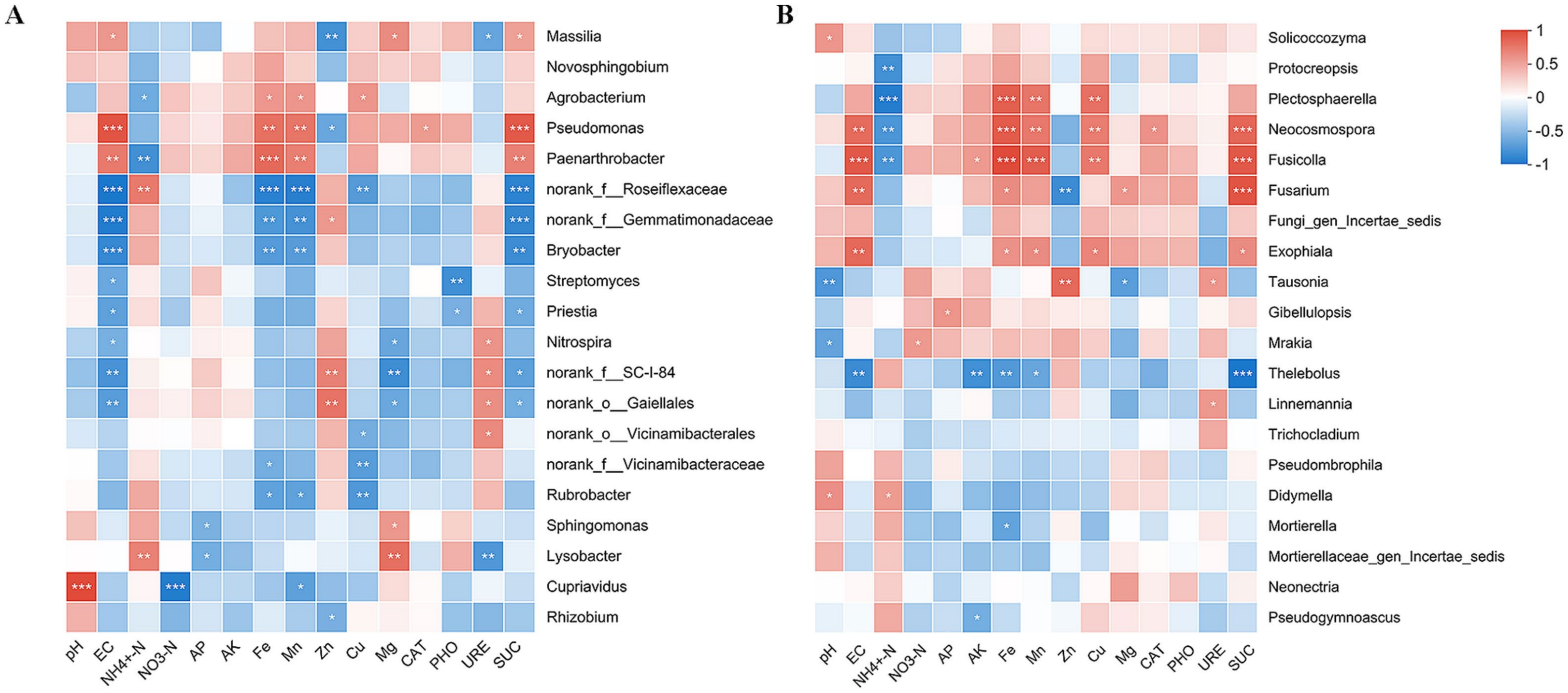
Spearman *correlation heatmap* showing relationships between soil environmental factors and dominant bacterial **(A)** and fungal **(B)** genera. Significance levels are indicated by asterisks: *0.01 < *p* ≤ 0.05, **0.001 < *p ≤* 0.01, and ***p ≤ 0.001.

Spearman correlation clustering heatmap analysis further clarified the relationships between soil environmental factors and key microbial taxa. Most dominant bacterial and fungal genera exhibited significant correlations with specific soil indices, indicating that the distribution and abundance of key members of both microbial communities were directly regulated by soil environmental conditions (Figure 6).

Further analysis demonstrated that most bacterial genera exhibited significant negative correlations with environmental factors (Figure 6A). Among these variables, soil EC, Fe, Mn, and SUC exerted particularly strong effects on bacterial community composition. Specifically, EC, Fe, Mn, and SUC were significantly negatively correlated with genera such as *norank_f_Roseiflexaceae*, *norank_f_Gemmatimonadaceae*, and *Bryobacter*, with EC showing particularly strong associations with these taxa. In contrast, *Pseudomonas* and *Paenarthrobacter* displayed significant positive correlations with these variables. *Pseudomonas* showed highly significant positive correlations with EC and SUC, whereas *Paenarthrobacter* exhibited a strong positive correlation with Fe. In addition, *Rubrobacter* and *norank_f_Vicinamibacteraceae* were significantly and negatively correlated with Fe and Cu. The genus *Streptomyces* was negatively influenced by EC and phosphatase activity (PHO). *Cupriavidus* exhibited a highly significant negative correlation with nitrate nitrogen (NO_3_^−^-N) but a highly significant positive correlation with pH. The genus *Lysobacter* was significantly positively associated with ammonium nitrogen (NH_4_^+^-N) and magnesium (Mg). *Massilia* also showed significant positive correlations with EC, Mg, and SUC. Overall, soil enzyme activities differed in their effects on bacterial communities, with SUC exerting a more pronounced influence than urease (URE), catalase (CAT), and phosphatase (PHO). For example, *Massilia* was significantly negatively correlated with URE but positively correlated with SUC.

In contrast, most fungal genera exhibited significant positive correlations with environmental factors (Figure 6B). Soil EC, Fe, Mn, Cu, and SUC exerted strong effects on fungal community composition, primarily showing positive effects. *Neocosmospora* and *Fusicolla* were significantly and positively correlated with all five factors. In particular, *Neocosmospora* presented strong positive correlations with Fe and SUC, whereas *Fusicolla* was strongly associated with EC, Fe, Mn, and SUC. Moreover, genera such as *Plectosphaerella* and *Fusarium* were significantly and positively correlated with multiple factors, including Fe, Mn, and Cu. In contrast, *Thelebolus* exhibited significant negative correlations with EC, AK, Fe, Mn, SUC, and other variables. NH_4_^+^-N was also negatively correlated with genera such as *Protocreopsis*, *Plectosphaerella*, *Neocosmospora*, and *Fusicolla*. Among soil enzymes, SUC had the strongest influence on fungal community structure. In summary, soil environmental factors predominantly exerted inhibitory effects on bacterial communities but generally promoted fungal communities. Fe, Mn, and SUC displayed the most pronounced correlations with rhizosphere microbial community composition.

### 3.6 Microbial community functional prediction

**Figure 7:**
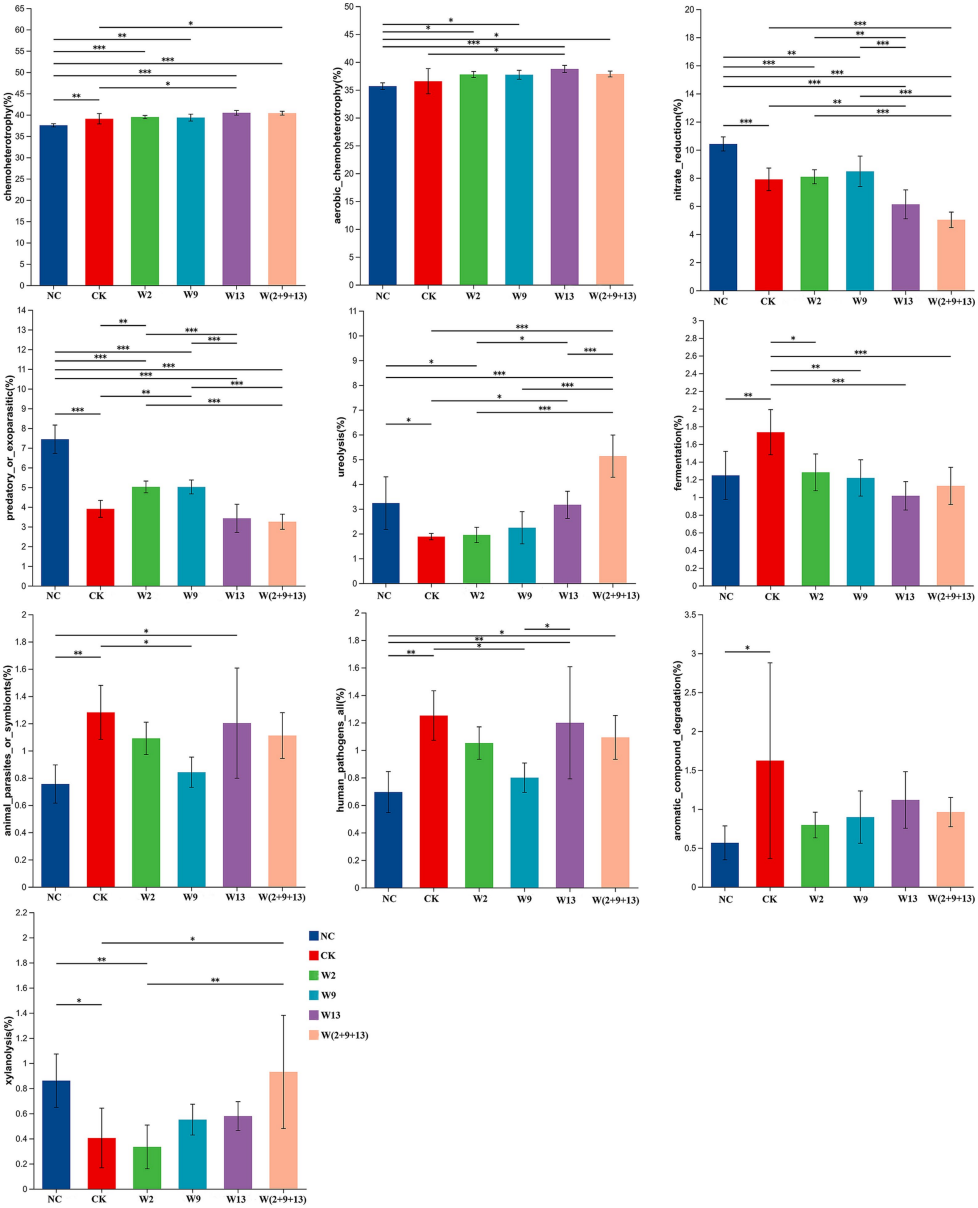
Enrichment of bacterial ecological functions under different treatments. Asterisks indicate statistically significant differences: *p ≤ 0.05 and **p ≤ 0.01.

The ecological functions of bacterial communities in sugar beet rhizosphere soil under different treatments were predicted using the FAPROTAX database. The ten most abundant predicted bacterial functional categories, based on the average relative abundance across samples, are presented in Figure 7. The results indicated that chemoheterotrophy and aerobic chemoheterotrophy dominated the predicted functional profiles across all treatment groups. In addition, functional groups associated with nitrate reduction, predatory or exoparasitic activity, ureolysis, and fermentation were also relatively abundant.

Significant differences in the relative abundance of bacterial functional groups were observed among treatments. Compared with the NC, CK exhibited a significantly higher abundance of chemoheterotrophy. Following the application of single or mixed Sphingomonad bacterial suspensions, the abundance of this functional group increased further and exceeded that observed in the CK group, with the differences between the W13 and W(2 + 9 + 13) treatments and CK reaching statistical significance. For aerobic chemoheterotrophy, no significant difference was detected between NC and CK. However, all bacterial suspension treatments showed significantly higher abundance than NC, whereas only the W13 treatment displayed a significant increase compared with CK. NC showed the significantly higher abundances of nitrate reduction and predatory or exoparasitic functions than all other treatments. Among the remaining treatments, W13 and W(2 + 9 + 13) exhibited the lowest nitrate reduction abundance, which was significantly lower than that of W2, W9, and CK. In contrast, W2 and W9 displayed the highest abundance of predatory or exoparasitic functions, significantly exceeding those observed in W13, W(2 + 9 + 13), and CK. The ureolysis function was most abundant in the W(2 + 9 + 13) treatment, demonstrating significant differences from all other groups, followed by W13, whereas CK exhibited the lowest abundance. For fermentation-related functions, both NC and all bacterial suspension treatments exhibited significantly lower abundance than CK, indicating an inhibitory effect of Sphingomonad on fermentation. Compared with NC, CK showed significantly higher abundance of functions associated with animal parasites or symbionts, human pathogens, and aromatic compound degradation. After Sphingomonad application, the abundances of these three functional categories declined in continuous cropping soil, with the W9 treatment showing the strongest inhibitory effects on the first two functions. Compared to NC, CK exhibited a significantly reduced abundance of xylanolysis. The W9, W13, and W(2 + 9 + 13) treatments significantly increased the abundance of this function, with W(2 + 9 + 13) showing the greatest increase, which was significantly higher than that of CK and approached the level observed in NC.

In summary, the three Sphingomonad strains and their mixed suspension exerted limited effects on the dominant chemoheterotrophy and aerobic chemoheterotrophy functions in the rhizosphere bacterial community but produced pronounced regulatory effects on several lower-abundance functional groups.

**Figure 8:**
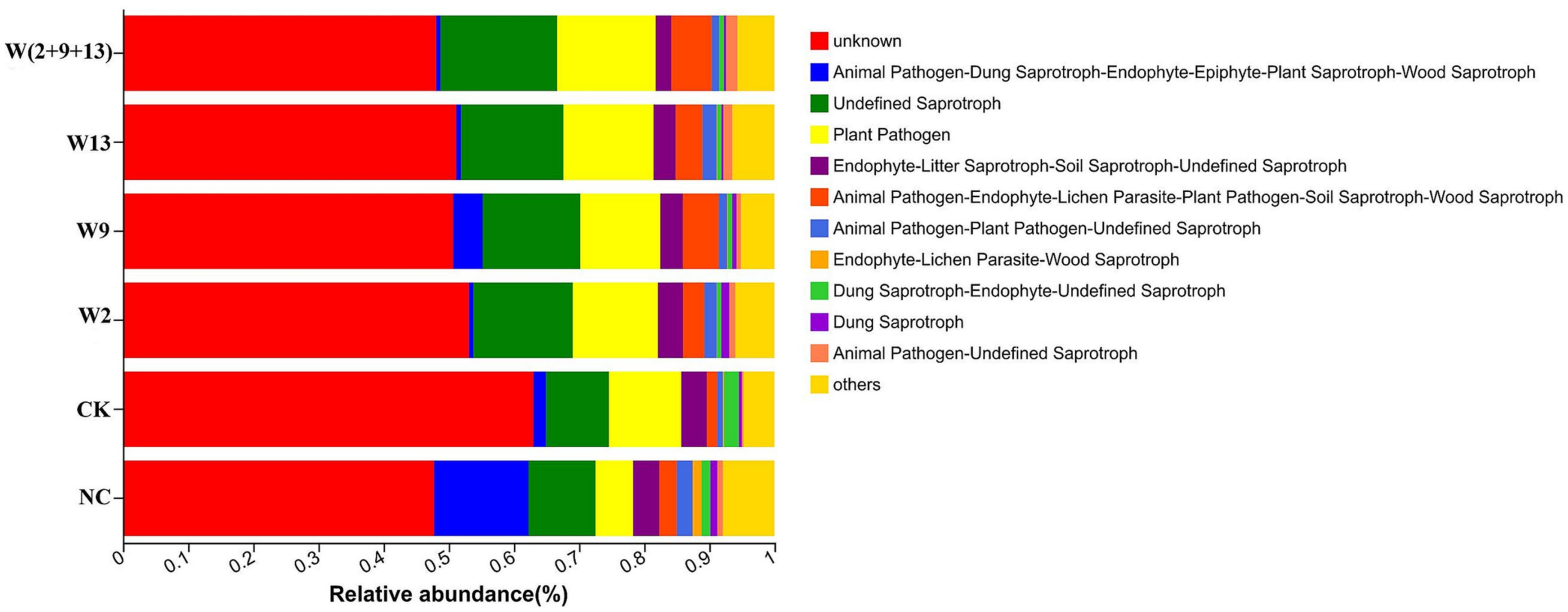
Relative abundance of fungal trophic types under different treatments.

Functional profiles of fungal communities in sugar beet rhizosphere soil were predicted under different treatment conditions using the FunGuild tool. The results showed that the relative abundances of fungal trophic functional types exhibited distinct distribution patterns among treatments (Figure 8). In the NC treatment, the three most abundant trophic types in descending order were the unknown fungal trophic type, Animal Pathogen–Dung Saprotroph–Endophyte–Epiphyte–Plant Saprotroph–Wood Saprotroph, and Undefined Saprotroph. In the remaining five treatments, the dominant trophic types were the unknown fungal trophic type (51.17–63.03%), Undefined Saprotroph (9.67–17.92%), and Plant Pathogen (11.12–15.13%). The CK group exhibited the highest relative abundance of unknown fungal trophic types. Furthermore, the NC treatment displayed the significantly higher relative abundances of Animal Pathogen–Dung Saprotroph–Endophyte–Epiphyte–Plant Saprotroph–Wood Saprotroph and Endophyte–Lichen Parasite–Wood Saprotroph than all other treatments, reaching 14.49 and 1.38%, respectively. Compared with CK, W9 treatment significantly increased the relative abundance of fungi assigned to Animal Pathogen–Dung Saprotroph–Endophyte–Epiphyte–Plant Saprotroph–Wood Saprotroph. In addition, all bacterial suspension treatments significantly increased the relative abundance of Animal Pathogen–Endophyte–Lichen Parasite–Plant Pathogen–Soil Saprotroph–Wood Saprotroph, while significantly reducing the relative abundance of Dung Saprotroph–Endophyte–Undefined Saprotroph, resulting in the levels similar to those observed in the NC treatment. Overall, these results indicated that the application of Sphingomonads primarily influenced the abundances of specific fungal functional groups, including Animal Pathogen–Endophyte–Lichen Parasite–Plant Pathogen–Soil Saprotroph–Wood Saprotroph and Dung Saprotroph–Endophyte–Undefined Saprotroph, while exerting relatively limited effects on other functional categories.

## 4 Discussion

PGPR can play a critical role in enhancing plant growth and mitigating continuous cropping constraints (Xu W. F. et al., 2023; Sun et al., 2024). In this study, three previously screened strains, including *Sphingobium abikonense* W2, *Sphingomonas panni* W9, and *Sphingomonas* sp. W13, were selected as inoculants for a systematic assessment of their impact on the growth performance of continuously cropped sugar beet seedlings, soil physicochemical properties, and rhizosphere microbial communities.

It is important to address the experimental design regarding the different inoculum concentrations used for strains W2, W9 and W13. While comparing treatments at identical cell densities is a common approach, our study aimed to compare the strains under their respective optimal application conditions. The concentrations used in the main experiment were determined through preliminary dose–response assays, which identified the specific density at which each strain achieved its maximum growth-promoting effect on sugar beet seedlings.

The finding that W9 required a 40-fold higher concentration than W13 to reach its optimal efficacy is, therefore, not a technical artifact but a biologically significant result. It suggests fundamental differences in the interaction mechanisms of these two strains with the host plant. The higher optimal density for W9 may reflect a lower efficiency in root colonization, a requirement for quorum sensing to activate beneficial functions, or a reduced competitive ability against the resident rhizosphere microbiome. In contrast, W13’ s efficacy at a lower density suggests a more efficient recognition or colonization strategy.

By comparing strains at their respective optima, we capture a more holistic picture of their potential as bio-inoculants: both the magnitude of the benefit (the ‘effect size’) and the ‘application cost’ (the required dose) are integral parts of a strain’s performance package. This approach provides a more realistic assessment for agricultural application, where the dose required to achieve a consistent effect is a critical economic and ecological consideration.

Following inoculation with strains W2, W9, W13, and their mixed suspension, the sugar beet seedlings exhibited significant increases in plant height, stem diameter, leaf fresh weight, and root fresh weight compared with the CK group. These growth parameters were restored to levels close to or even exceeding those observed under the NC. This response was consistent with the growth-promoting effects reported for *Sphingomonas* inoculation in maize (Wang F. et al., 2022). In contrast, the growth-promoting effect of the Sphingomonads suspension in our study substantially outperformed that of *Bacillus*, a plant growth-promoting rhizobacterium which, when applied to sugar beet, increased seedling fresh weight by only 31.08–91.55% (Sun Y. C. et al., 2025; Sun et al., 2025). Long-term monoculture is known to induce marked alterations in soil physicochemical properties (Chen et al., 2020). Our results indicate both shared and strain-specific effects of Sphingomonad on soil properties. The consistent increase in pH and sucrase activity implied a general enhancement in soil metabolic activity. However, the distinct profiles of AP, AK, phosphatase, and urease activities highlight the functional differentiation among individual strains. Notably, the mixed-strain treatment did not exhibit a simple additive effect of the individual strains, suggesting potential inter-strain interactions that modulate the expression of these specific soil functions. This response pattern was comparable to the effects reported when *Pseudomonas fluorescens* MC46 was applied as a PGPR in pot experiments (Sipahutar et al., 2018). Overall, the three Sphingomonad strains significantly promoted the growth of sugar beet seedlings under continuous cropping conditions and effectively modulated key soil physicochemical properties, supporting their potential application for alleviating continuous cropping constraints.

Recent studies have demonstrated that PGPR can indirectly enhance plant productivity by reshaping the soil microbial community (Hu et al., 2021; Wang F. et al., 2022; Vuolo et al., 2022). In this study, high-throughput sequencing was employed to characterize rhizosphere microbial communities of continuously cropped sugar beet seedlings before and after Sphingomonad application. Both alpha- and beta-diversity analyses indicated that the Sphingomonad treatments markedly altered the composition and structure of bacterial and fungal communities in the rhizosphere under continuous cropping conditions. Among the treatments, W2, W9, W13, and their mixture increased fungal diversity and richness to varying extents, which is consistent with the trends reported for the *Sphingomonas* strain Hbc-6 (Wang et al., 2024). In contrast, the responses of bacterial diversity and richness differed from some previous findings (Taswar et al., 2023). This discrepancy may be attributed to the rapid proliferation of Sphingomonads, which can compete with indigenous bacteria for limited rhizosphere resources, thereby suppressing the growth of certain native bacterial populations (Liu et al., 2022).

Analysis of rhizosphere microbial composition indicated that Pseudomonadota, Actinomycetota, Acidobacteriota, Bacteroidota, and Chloroflexota were the dominant bacterial phyla in sugar beet rhizosphere soil. Notably, Pseudomonadota consistently exhibited the highest relative abundance, confirming its dominant status, a pattern also widely reported in the rhizospheres of blueberry and ginger (Wang et al., 2023; Wang and Yang, 2024). Following Sphingomonad inoculation, the relative abundance of Pseudomonadota increased significantly, with the mixed bacterial suspension treatment exhibiting the strongest response, even exceeding that observed in the NC. This may reflect a direct synergistic growth advantage among the inoculated Sphingomonad strains; alternatively, it may represent a broader, indirect community-level response facilitated by their combined activity. This finding is consistent with previous studies, which indicate that microbial inoculants can significantly alter the composition and function of soil microbiota (Sun Y. C. et al., 2025; Sun X. L. et al., 2025). In addition, Bacteroidota associated with potential biocontrol functions (Zheng et al., 2020) were promoted. At the bacterial genus level, *Pseudomonas*, *Sphingomonas*, *Cupriavidus*, *Massilia*, and *Novosphingobium* were the predominant genera across treatments. Inoculation with Sphingomonads increased the relative abundances of these taxa, particularly *Pseudomonas*, whose abundance surpassed that of the NC group. Similar enrichment patterns have been documented in previous studies involving PGPR (Song et al., 2021). Existing evidence suggests that beneficial rhizobacteria (e.g., *Pseudomonas*, *Sphingomonas*, *Cupriavidus*, and *Massilia*) can enhance the rhizosphere microenvironment through the production of volatile organic compounds and secondary metabolites, regulation of plant hormone balance, and participation in root signal transduction, thereby promoting plant growth under biotic or abiotic stress conditions (Yu et al., 2021; Lombardino et al., 2022; Luo et al., 2024; Yuan et al., 2024).

However, a substantial proportion of *Sphingomonas* was also detected in the non-inoculated CK and NC soils, yet these treatments did not exhibit comparable plant growth promotion. This discrepancy suggests that the beneficial effects are not simply due to increased genus-level abundance, but rather to the introduction of specific, functionally superior strains (W2, W9, W13). Compared to the indigenous *Sphingomonas* population, these inoculated strains–originally isolated from the same soil ecosystem–likely possess enhanced functional traits (e.g., metabolic versatility or plant growth-promoting capabilities) that enable them to occupy key rhizosphere niches and potentially outcompete or functionally complement the native population. Crucially, inoculation with these strains appeared to drive a broader community shift, as evidenced by the concurrent enrichment of other beneficial taxa. Notably, the relative abundance of *Pseudomonas*–a genus known for its plant-beneficial functions–was higher in the NC (rotation) soil than in the CK (continuous cropping) soil, and increased further upon inoculation, with the mixed consortium showing the highest enrichment. This suggests that the introduced Sphingomonads strains may facilitate cooperative or supportive interactions with other beneficial taxa, thereby contributing to the observed improvements. Such strain-specific, function-driven restructuring of the microbial community toward a more plant-beneficial configuration (Chi et al., 2025; Li et al., 2025; Liu et al., 2025; Mawarda et al., 2020; Pereira et al., 2025; Sun et al., 2022; Sun Y. C. et al., 2025; Sun X. L. et al., 2025) is further substantiated by broader shifts in overall microbial composition observed in the inoculated treatments.

Within the fungal community, Basidiomycota and Ascomycota were the dominant phyla, consistent with observations reported for tomato rhizospheres treated with Trichoderma-based fertilizers (Pang et al., 2017). Sphingomonad inoculation shifted the fungal community structure by generally reducing the relative abundance of Basidiomycota and increasing that of Ascomycota. Within this broader shift, the enrichment of specific genera, such as *Fusarium* and *Neocosmospora*, was observed, which include species known to be plant pathogens. However, this enrichment does not necessarily imply an increased disease risk. Instead, it may reflect a state of “productive tension” within a more complex and resilient soil microbiome. First, the relative abundances of these genera remained low, and they did not emerge as dominant taxa, suggesting a limited ecological influence under the experimental conditions. Second, pathogenicity within these genera is strain-specific, and not all members are virulent. Importantly, the concurrent enrichment of beneficial bacteria and the overall improvement in plant health indices observed in this study suggest that any potential pathogenic effects were likely mitigated by the inoculated beneficial consortium, possibly through mechanisms such as competitive exclusion or induced systemic resistance.

This study employed LEfSe to investigate the effects of different treatments (crop rotation (NC), continuous cropping (CK), and various Sphingomonads inoculation treatments) on the rhizosphere microbial community structure of sugar beet. The results demonstrated distinct response patterns of bacterial and fungal communities to continuous cropping obstacles and microbial inoculation. Notably, the mixed inoculant treatment (W(2 + 9 + 13)) exhibited a greater capacity to modulate the fungal community, consistent with previous studies on rhizosphere microbial assembly and functional regulation. The pronounced differences in the dominant taxa between the rotation (NC) and continuous cropping (CK) control groups reflect the profound influence of agricultural management practices on rhizosphere microecology. The enrichment of oligotrophic or complex organic matter-degrading taxa in the CK group, such as Acidobacteriota and Chloroflexota, may be related to the prolonged accumulation of root exudates and alterations in soil organic matter composition under long-term monocropping (Fierer, 2017). In contrast, the taxa enriched in the NC group, including Bacteroidota, *Rubrobacter*, and various rhizobia-associated groups, are generally linked to higher soil nutrient availability and more favorable plant–microbe interactions. These findings support the role of crop rotation in maintaining soil microecological balance and promoting the proliferation of beneficial microorganisms (Philippot et al., 2013). Following Sphingomonad inoculation, particularly under single-strain treatments, the number of bacterial biomarker taxa decreased. This trend likely reflects the selective pressure exerted by introduced exogenous microorganisms on the indigenous bacterial community, leading to the suppression of certain native taxa and a consequent simplification of the community structure. For example, the enrichment of *Nitrospira* and Bdellovibrionota in the W9 treatment, which are involved in nitrogen cycling and bacterial parasitism, respectively, suggests that the inoculant treatment may have influenced soil nitrogen transformation and microbial predatory interactions (Daims et al., 2015). Notably, the mixed inoculant treatment [W(2 + 9 + 13)] resulted in the enrichment of a greater number of fungal biomarker taxa, including potentially plant growth-promoting or pathogen-suppressing genera, such as *Hannaella* and *Plectosphaerella* (Shah et al., 2021). This observation indicates that the mixed inoculant, potentially through synergistic interactions, reshapes the fungal community structure and enhances its ecological functional diversity more effectively, thereby contributing to the alleviation of continuous cropping obstacles. From a biomarker perspective, these results highlight the potential of Sphingomonad inoculants, particularly the mixed formulation, to modulate rhizosphere microbial communities, with a pronounced impact on fungal community structure in continuous-cropping soils. However, whether changes in microbial biomarkers are directly linked to improved soil functions (such as disease suppression and nutrient transformation) and enhanced sugar beet yield still requires further validation through long-term field trials and integrative multi-omics analyses (Toju et al., 2018).

The response of rhizosphere soil microbial communities to environmental factors can directly regulate the abundance and composition of plant-associated microorganisms, thereby ultimately influencing host plant growth and health. In this study, Mantel tests and Spearman correlation analyses were jointly applied to systematically elucidate the multiscale effects of environmental factors on microbial communities at both the overall community structure and key taxon levels. At the community scale, fungal assemblages exhibited stronger environmental dependence than bacterial assemblages. In contrast, at the key species level, the dominant taxa of both bacteria and fungi demonstrated extensive and significant associations with environmental variables. In contrast to the community-level pattern, analysis of the dominant bacterial genera revealed that most of these key taxa were negatively correlated with factors such as EC, Fe, Mn, and SUC. This pattern differs from the findings reported by Chaudhary et al. They observed that PGPR increased soil iron availability (Doongar et al., 2019). This discrepancy may be attributed to the specific soil conditions in the present study, where the interactions among multiple ions, including Fe and Mn, may have generated toxic effects that inhibited the growth of most bacterial taxa (Xu C. et al., 2023). Conversely, certain bacterial groups (e.g., *Pseudomonas* and *Paenarthrobacter*) exhibited significant positive correlations with these factors, indicating a higher degree of ecological adaptability. Furthermore, the positive association between URE activity and *Nitrospira* was consistent with the established mechanism by which arbuscular mycorrhizal fungi and PGPR synergistically promote nutrient cycling (Yu et al., 2022). In contrast to the overall suppression observed in bacterial communities, several fungal taxa, including *Neocosmospora*, *Fusicolla*, *Plectosphaerella*, and *Fusarium*, exhibited significant positive correlations with multiple environmental variables such as Fe, Mn, Cu, and SUC. Similar response patterns of fungal communities to environmental drivers have been reported in previous studies (Li J. X. et al., 2023; Li N. et al., 2023; Jiang et al., 2024; Chi et al., 2025). In summary, these results propose a potential ecological strategy whereby the stability of bacterial communities can be maintained through internal functional redundancy and the sustained activity of a limited number of key taxa. Although the bacterial community structure exhibited weaker macro-scale responses to environmental changes than fungal communities, the core functional groups responded rapidly and directly to environmental disturbances, thereby contributing to the maintenance of overall ecosystem functional stability.

Functional prediction analyses based on the FAPROTAX and FunGuild databases further demonstrated that the introduction of Sphingomonad systematically reshaped the microbial functional profiles of sugar beet rhizosphere soil, markedly altering both bacterial metabolic pathways and fungal trophic structures. In bacterial functional predictions, the inoculation treatments exerted limited effects on dominant core functions, such as chemoheterotrophy, but significantly regulated several specific functional groups with clear ecological relevance. Notably, functions directly associated with nutrient cycling, including ureolysis and xylanolysis, were significantly enhanced, indicating improved rhizosphere carbon and nitrogen turnover. Concurrently, bacterial suspension treatments significantly suppressed functional groups associated with potential health risks, such as animal parasites, symbionts, and human pathogens, suggesting a reduced ecological risk in continuously cropped soils. In fungal functional predictions, inoculation shifted the functional composition of fungal communities towards patterns characteristic of NC. For instance, multifunctional trophic groups (e.g., Animal Pathogen–Dung Saprotroph–Endophyte–Epiphyte–Plant Saprotroph–Wood Saprotroph) that are typically more abundant under rotation systems were significantly restored in the W9 treatment. In addition, rotation-associated functional groups, including Endophyte–Lichen Parasite–Wood Saprotroph, exhibited increasing trends following inoculation. Collectively, these results indicate that Sphingomonad inoculation contributed to the reconstruction of a rhizosphere micro-ecosystem with greater structural complexity and functional stability, consistent with conclusions reported in previous studies (Mesquita Cunha et al., 2024; Qiao et al., 2024; Chi et al., 2025).

Inoculation with Sphingomonad strains W2, W9, and W13 directly promoted sugar beet seedling growth and improved the physicochemical properties of continuously cropped soil, including increases in soil pH and key enzyme activities. These strains exerted an indirect regulatory effect by systematically reshaping the rhizosphere micro-ecosystem. The underlying mechanisms can be summarized in three aspects. First, the microbial community structure was optimized through the selective enrichment of beneficial bacteria such as *Pseudomonas* and the redirection of fungal community composition towards a state resembling that of healthy crop rotation soil. Second, ecological functions were strengthened, as evidenced by significant enhancements in critical carbon and nitrogen cycling processes, including ureolysis and xylanolysis, along with effective suppression of pathogen-associated functions. Third, environmental adaptability was enhanced, as the key microbial taxa (e.g., *Pseudomonas*) exhibited pronounced positive tolerance to ionic stresses such as Fe and Mn. Collectively, this study elucidated the mechanism by which Sphingomonad strains alleviated continuous cropping obstacles through a cascading pathway involving soil property improvement, microbial community regulation, and functional optimization, thereby providing a theoretical basis and valuable microbial resources for the development of targeted microbial fertilizer products.

## Data Availability

The datasets generated for this study can be found in the NCBI Sequence Read Archive (SRA) database under accession numbers PRJNA1404148 (ITS) and PRJNA1403766 (16S).

## Footnotes

1
http://www.mothur.org/wiki/Calculators

2
http://huttenhower.sph.harvard.edu/LEfSe

3
https://cloud.majorbio.com
